# Patients’ and health professionals’ attitudes and perceptions towards the initiation of preventive drugs for primary prevention of cardiovascular disease: a systematic review of qualitative studies

**DOI:** 10.3399/bjgpopen20X101087

**Published:** 2020-10-21

**Authors:** Olla Qadi, Nakawala Lufumpa, Nicola Adderley, Danai Bem, Tom Marshall, Farina Kokab

**Affiliations:** 1 PhD student, Institute of Applied Health Research, University of Birmingham, Birmingham, UK; 2 Lecturer in Health Informatics and Epidemiology, Institute of Applied Health Research, University of Birmingham, Birmingham, UK; 3 Honorary Research Fellow, Institute of Applied Health Research, University of Birmingham, Birmingham, UK; 4 Professor of Public Health & Primary Care, Institute of Applied Health Research, University of Birmingham, Birmingham, UK; 5 Research Fellow, Institute of Applied Health Research, University of Birmingham, Birmingham, UK

**Keywords:** Qualitative research, cardiovascular disease, statins, antihypertensive drugs, primary prevention

## Abstract

**Background:**

Statins and antihypertensive agents are recommended for primary prevention of cardiovascular disease (CVD), but they are not always prescribed to eligible patients.

**Design & setting:**

A systematic review of qualitative studies.

**Aim:**

To explore health professionals’ and patients’ attitudes towards cardiovascular preventive drugs.

**Method:**

MEDLINE, Embase, PsychINFO, CINAHL, ASSIA, HMIC, Conference Proceedings Citation Index, and Open Grey were searched for studies of qualitative design without restrictions on date or language. Two reviewers performed study selection, data extraction, quality assessment, and thematic synthesis.

**Results:**

In total, 2585 titles and abstracts were screened, yielding 27 studies, of which five met eligibility criteria on full text assessment. These included 62 patients and 47 health professionals. Five themes emerged about patient attitudes: questioning preventive drugs; perceived benefit and risks, such as improving quality of life; patient preferences; trust in health professional judgement; and family, friends, and media influences. Five themes emerged about health professional attitudes: addressing patient concerns and information; duty as a health professional to prescribe; uncertainty about preventive drug prescribing; recognising consequences of prescribing, such as unnecessary medicalisation; and personalised treatment.

**Conclusion:**

The attitudes of patients and health professionals regarding drug initiation for primary prevention reflect the complexity of the patient–health professional encounter in primary practice. For prescribing to be more adherent to guidelines, research should further investigate the patient–health professional relationship and the appropriate communication methods required when discussing drug initiation, specifically for primary prevention.

## How this fits in

Cardiovascular drugs for primary prevention are not always prescribed to eligible patients. This review explored the attitudes towards preventive drugs from both the patients' and health professionals' perspectives. The findings will add to previous knowledge about barriers to preventive drug prescribing, specifically for primary prevention. Identifying these attitudes may improve the decision-making process and provide health professionals with an understanding of what can influence the decision to initiate cardiovascular drugs for primary prevention.

## Introduction

CVD is the leading cause of death worldwide.^1^ Statins for primary prevention of CVD reduce the risk of cardiovascular events by 25%.^2–4^ The use of antihypertensive drugs to lower blood pressure in patients with systolic blood pressure ≥140 mmHg is associated with a reduction in major cardiovascular events by 12%.^5^ The National Institute for Health and Care Excellence (NICE) recommends statins for primary prevention of CVD in patients with a 10-year CVD risk of ≥10%.^6^ NICE recommends antihypertensive drugs for patients <80 years with blood pressure ≥140/90 mmHg and a 10-year CVD risk of ≥10.^7^ Antihypertensive drugs are also recommended for adults of any age with blood pressure ≥160/100 mmHg.^7^ Despite the recommendations, patients at high risk of CVD are not always initiated on statins.^8,9^ A study using data from primary care settings in the UK estimated that a statin was prescribed in 49.7% of patients with ≥20% CVD risk, and 19.2% of patients with 10–19% CVD risk.^9^ Suboptimal initiation of hypertension treatment was reported by a number of studies.^10–12^ In the US, a retrospective analysis of electronic medical records of 10 022 adults with incident hypertension (defined as blood pressure ≥140/90 mmHg) and no previous antihypertensive prescription from a large primary care practice found 34% of hypertensive patients aged 18–39 years, and 44% aged 40–59 years, were initiated on antihypertensive drugs.^10^ Another study analysed data from health examination surveys across 20 countries and reported that for patients aged 30–84 years with systolic blood pressure of ≥140 mmHg, 62% were diagnosed with hypertension and 53% were treated in the UK, compared to 85% diagnosed and 80% treated in the US.^13^ One study using data from a national survey of primary care visits of patients diagnosed with hypertension in the US found that treatment was initiated in only 26.4% of visits.^14^

Health professionals may not initiate treatment because they feel uncertain about the appropriate time to do so, especially in hypertensive patients.^15^ They may lack knowledge about the guidelines, or have concerns about over-medicalisation of healthy individuals, patient compliance, and side effects.^16–19^ Patients may decline treatment for a number of reasons including: preferring to avoid medication; preference for an alternative, such as lifestyle modification; concerns about their GP’s medical judgement; or potential side effects.^18^ Quantitative studies provide evidence of patient factors that predict drug initiation such as diabetes and increasing age.^20,21^ However, many important factors influencing the drug initiation decision are unrecorded, and these studies do not answer the question of how health professionals and patients reach the drug initiation decision. A qualitative approach will provide additional insight into the drug initiation decision.

Two recent qualitative systematic reviews described GPs’ perspectives on the prevention of CVD and attitudes of patients towards statins.^22,23^ Themes relevant to GPs’ perspectives on preventive drug prescribing were: duty to prescribe medication; ascertaining patients’ drive for lifestyle change; and avoiding over-medicalisation.^22^ Patient-related themes identified regarding statin uptake were confidence in prevention; medical distrust (scepticism about over-prescribing; pressure to start therapy); and threat to health (debilitating side effects).^23^ Both reviews were conducted by the same team and explored the views of patients and GPs separately. Neither review could identify if the reported themes related specifically to primary or secondary prevention, and were restricted to studies in the English language. The reasons behind initiating drugs can differ between patients who have experienced a CVD event and those who have not. Therefore, the need was recognised for a review that specifically explores drug initiation for primary prevention from both the patients’ and health professionals’ perspectives simultaneously, to give a better understanding of the decision-making process.

The aim was to determine the attitudes of health professionals and patients towards cardiovascular preventive drugs, and generate information about factors that influence drug initiation in primary care settings. The findings will inform future quantitative research of factors associated with prescribing cardiovascular drugs for primary prevention. In addition, they will provide health professionals, policymakers, and the public with an understanding of the facilitators and barriers of preventive drug prescribing in primary care.

## Method

This systematic review follows the reporting guidelines of the enhancing transparency in reporting the synthesis of qualitative research statement and the Preferred Reporting Items for Systematic Reviews and Meta-Analyses (PRISMA).^24,25^ The protocol for this systematic review is registered with PROSPERO and published in BMJ Open.^26^ The search strategy was developed using the Sample, Phenomenon of Interest, Design, Evaluation, Research type (SPIDER) framework.^27^ The components of SPIDER are detailed in Table 1 and outline which studies are eligible for inclusion in this review. Detailed inclusion and exclusion eligibility criteria were discussed in the protocol paper.^26^ The search strategy was initially formulated for the MEDLINE database and adapted for other databases (EMBASE, PsychINFO, CINAHL, Applied Social Sciences Index and Abstracts [ASSIA], Conference Proceedings Citation Index [Web of Science], Healthcare Management Information Consortium [HMIC], and Open Grey). The literature search captured qualitative studies (including mixed methods studies) from database inception to September 2018. A sample search strategy can be found in Supplementary Table S1. Two independent reviewers (OQ and NL) screened the titles and abstracts of studies retrieved from databases. Studies that met the eligibility criteria were selected for full text assessment. Articles included were then assessed for their full text content. Any disagreements between reviewers were resolved by discussion, or referred to a third independent reviewer in cases where the two reviewers failed to reach consensus.

**Table 1.** Summary of Sample, Phenomenon of Interest, Design, Evaluation, Research (SPIDER) framework

**S**ample	Health professionals (GPs or nurse practitioners) who prescribe statins or antihypertensive drugs. Patients eligible for cardiovascular preventive drugs or offered a prescription of statin or an antihypertensive drug for primary prevention of cardiovascular disease.
**P**henomenon of **I**nterest	The initiation or prescription of statins or antihypertensive drugs.
**D**esign	Studies including qualitative data collection or analysis methods.
**E**valuation	Attitudes, perceptions, views, or experiences of health professionals or patients related to the initiation of cardiovascular preventive drugs for primary prevention.
**R**esearch type	Qualitative and mixed methods studies.

### Data extraction and quality assessment

All relevant text and quotations under the results and conclusion section in each study was extracted and recorded in NVivo (version 12) software to facilitate data analysis. The quality of included studies was appraised using the Critical Appraisal Skills Programme (CASP) Qualitative Research Checklist.^28^ The Consolidated Criteria for Reporting Qualitative Research (COREQ) checklist was used to assess the comprehensiveness of reporting in included studies.^29^ The data extraction and quality assessment forms were discussed by two independent reviewers (OQ and NL) and any disagreements were resolved through discussion.

### Data synthesis and analysis

The method of thematic synthesis was adopted, where themes are constructed through an inductive process to answer the review question.^30^ Two reviewers independently coded each study line by line. Codes were organised into descriptive themes. Similar concepts were then grouped to develop analytical themes. The reviewers read all the codes and discussed them to ensure that the emergent themes captured all the information reported by the primary studies (OQ and NL). Disagreements about the themes were discussed and adjusted accordingly. The final themes were discussed with an advisory group to ensure appropriateness (OQ, TM, NA, and FK).

## Results

**Figure 1.**
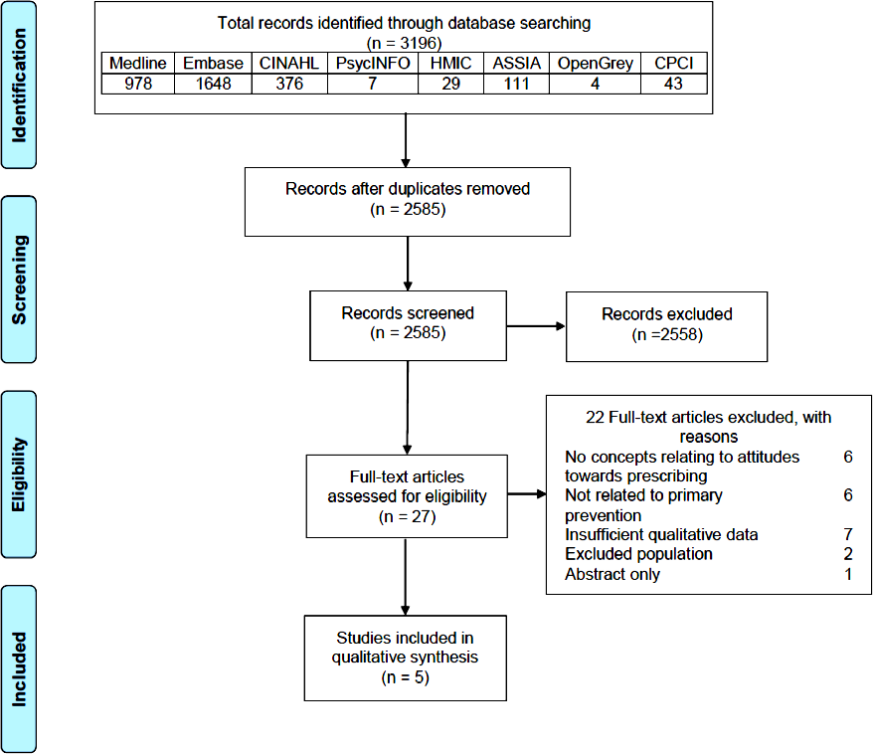
PRISMA flow diagram

The databases searches retrieved a total of 3196 records. After removing duplicates, 2585 titles and abstracts were screened for eligibility. During the screening phase, 2558 studies were excluded based on title and abstract. Of the remaining 27 studies, 22 were excluded based on full text assessment, leaving five studies. The five studies included 47 health professionals (42 GPs and five practice nurses) and 62 patients. The majority of studies utilised face to face interviews and analysed their data using thematic analysis. Studies were excluded mainly due to the lack of differentiation between primary and secondary prevention, concepts not relating to attitudes towards prescribing, and lack of qualitative output. The characteristics of excluded full text studies and reasons for exclusion are presented in Supplementary Table S2. Screening the reference lists of included studies revealed no additional eligible studies. A PRISMA flow diagram detailing the selection process is presented in Figure 1. The characteristics of included studies are summarised in Table 2. A detailed description of included studies is provided in Supplementary Table S3.

**Table 2.** Characteristics of included studies

**Authors**	**Study title**	**Cardiovascular drug**	**Health professionals,** ***n***	**Patients,** ***n***	**Study design**	**Data collection**	**Data analysis**	**Quality of study**
**UK**
**Gale, 2011** ^18^	Attitudes of patients and GPs towards medication for primary prevention of CVD after they had received detailed information about CVD risk and the absolute benefits of preventive medicine.	Cardiovascular preventive drugs (statin and antihypertensive drugs)	13	17	Qualitative	**Patients:** face to face interviews**GPs:** interviews	Thematic analysis	Good
**Turner, 2013** ^34^	Reasons for the variation in statin uptake by high risk patients	Statin	4	28	Qualitative	**Patients:** 10 minutes telephone interviews.**GPs:** 30 minutes face to face interviews.	Thematic analysis	Fair
**Virdee, 2013** ^32^	Primary care physicians’ and practice nurses’ attitude towards using the polypill for CVD prevention	Polypill	16 (11 physicians, five practice nurses)	–	Qualitative	Semi-structured interviews and transcribed verbatim.	Thematic analysis.	Good
**Virdee, 2015** ^33^	Patient attitudes about the use of a polypill for CVD prevention	Polypill	–	17	Qualitative	Semi-structured interviews.	Thematic analysis.	Good
**Sweden**								
**Hultberg, 2012** ^31^	GPs’ descriptions of their thoughts and actions when prescribing cardiovascular preventive drugs	Cardiovascular preventive drugs (no mention of specific drugs)	14	–	Qualitative	Group interviews of GPs practising together.	Qualitative content analysis.	Good

CVD cardiovascular disease

### Assessment of quality and reporting

All included studies addressed most CASP criteria items, stating their aims, data collection and analysis methods, findings, and ethical issues. However, four studies did not discuss a CASP item regarding researchers critically examining their role and potential bias during the research process. The comprehensiveness of reporting in included studies varied. All studies reported the participant selection process and the study setting. Almost all studies reported data saturation.^18,31–33^ Details of the comprehensiveness of reporting by the included studies, using the COREQ checklist, are presented in Table 3.

**Table 3.** COREQ checklist detailing studies’ reporting of each item

**Item**	**Studies reporting item** ^a^	**Studies reporting item, *n* (%)**
**Domain 1: Research team and reﬂexivity**		
*Personal characteristics*		
Interviewer/facilitator	(18, 31–33)	4 (80)
Credentials	(33)	1 (20)
Occupation	(33)	1 (20)
Sex	–	0
Experience and training	(33)	1 (20)
*Relationship with participants*		
Relationship established	–	0
Participant knowledge of the interviewer	–	0
Interviewer characteristics	–	0
**Domain 2: study design**		
*Theoretical framework*		
Methodological orientation and theory	(31–34)	4 (80)
*Participant selection*		
Sampling	(18, 31–34)	5 (100)
Method of approach	(18, 31–34)	5 (100)
Sample size	(18, 31–34)	5 (100)
Non-participation	(32–34)	3 (60)
*Setting*		
Setting of data collection	(18, 31–34)	5 (100)
Presence of non-participants	–	0
Description of sample	(18, 31–34)	5 (100)
*Data collection*		
Interview guide	(18, 31–34)	5 (100)
Repeat interviews	(18, 31)	2 (40)
Audio/visual recording	(18, 31–34)	5(100)
Field notes	(31–34)	4 (80)
Duration	(32–34)	3 (60)
Data saturation	(18, 31–33)	4 (80)
Transcripts returned	(32, 33)	2 (40)
**Domain 3: analysis and ﬁndings**		
*Data analysis*		
Number of data coders	(18, 31–33)	4 (80)
Description of the coding tree	(31–33)	3 (60)
Derivation of themes	(18, 31–34)	5 (100)
Software	(32, 33)	2 (40)
Participant checking	–	0
*Reporting*		
Quotations presented	(18, 31–34)	5 (100)
Data and ﬁndings consistent	(18, 31–34)	5 (100)
Clarity of major themes	(18, 31–34)	5 (100)
Clarity of minor themes	(18, 31–34)	5 (100)

^a^Studies are identified by their reference number.^18,31–34^

COREQ Consolidated Criteria for Reporting Qualitative Research

### Thematic synthesis

**Figure 2.**
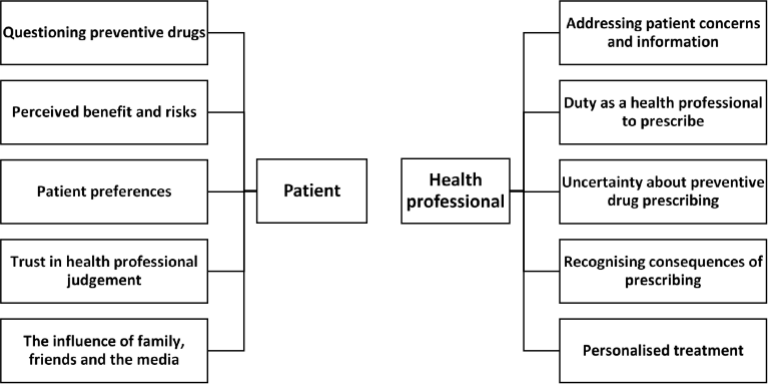
Diagram of patient and health professional related themes

Five themes emerged relating to patient attitudes: questioning preventive drugs; perceived benefits and risks; patient preferences; trust in health professionals' judgement; and family, friends, and media influences. Five themes emerged about health professional attitudes: addressing patient concerns and information; duty as a health professional to prescribe; uncertainty about preventive drug prescribing; recognising consequences of prescribing; and personalised treatment. A diagram of the main themes is presented in Figure 2. A comprehensive table of quotations to illustrate each theme is presented in Supplementary Table S4.

#### Patient attitudes towards preventive drugs

##### Questioning preventive drugs

###### Scepticism due to changes in guideline recommendations

Patients expressed lack of confidence in preventive drugs. One patient referred to the change in aspirin guidelines relating to primary prevention (it is no longer recommended) and felt this change could apply to other drugs, leading them to lose trust in their healthcare professional.^33^

###### Mistrust in research, academia, and the pharmaceutical industry

Some patients expressed their lack of belief in research findings about the effectiveness of cardiovascular preventive drugs, expressing mistrust in academia and the pharmaceutical industry,^18^ referring to the profit motive and questioning the validity of the information given by academic researchers.^18^

###### Unknown side effects and dependency

Patients expressed concerns about adverse effects, mentioning the possibility of unknown side effects and drug dependency.^18^ Some patients declined treatment because of concerns about side effects, whereas others were willing to take treatments despite any possible risks.^18,34^ Similar concerns were raised by patients about the polypill, one describing it as a *‘foreign body*’ that can create problems.^33^

###### Enabling patient compliance with a healthy lifestyle

Some patients expressed their reservations about taking a polypill for primary prevention and explained that the idea of taking a tablet to prevent disease could cause individuals to be reliant on the medication and complacent about healthy eating and exercise.^33^

###### Necessity

Willingness to accept preventive drugs was linked to perceived necessity. Patients were willing to take preventive drugs if they felt it was necessary because of a life-threatening condition, as a last option for a severe condition, or if it was recommended by their healthcare provider.^18,34^ Some patients considered that taking medication in the absence of a condition is unnecessary and leads to medicalising healthy individuals.^33^ Patients discussing the polypill expressed that a blanket approach where a drug is prescribed for everyone over a specific age is concerning. However, they justified the need for medication if the patient has a high-risk condition, such as high blood pressure and cholesterol.^33^

##### Perceived benefit and risks

###### Perceived risk

Patients’ perceived risk might influence their willingness to accept drugs.^18,33^ One patient expressed that his perception of risk depends on the severity of the condition, mentioning that conditions such as diabetes would worry him more than others and possibly prompt action.^18^

###### Rationalising the benefit

Willingness to take preventive drugs depends on how much patients believe the benefits outweigh the inconveniences; for example, if the drug extended lifespan and improved quality of life or lowered risk of having a heart attack.^18,34^ Some patients considered prevention to be better than a cure.^18,33^ A few patients discussing the polypill considered the financial benefits of prevention to the NHS.^33^

##### Patient preferences

Some patients expressed their preference to avoid medication or trying natural approaches, such as alternative medicine, and believed in the body’s capacity to heal itself.^18^ Patients preferred to attempt lifestyle modification before agreeing to take medication.^18,34^ Patients that were not on statins reported that they decided with their health professional to try lifestyle modifications first. They felt that their health professional supported their preference for lifestyle changes.^34^

##### Trust in health professionals' judgement

Patients discussing their attitudes towards preventive drugs explained that they would follow what their health professional recommended. This was related to their trust in the health professional and their expertise.^18^ Trust in their health professional was a reason for accepting treatment with statins.^34^ One study discussed GPs’ recommendations as an influential factor in a patient’s decision to accept.^18^

##### The influence of family, friends, and the media

Some patients explained that their attitude towards preventive drugs is influenced by similar experiences of family and friends. Family and friends’ experiences of side effects led patients to question preventive drugs,^18^ whereas experiences such as death from heart attacks influenced patients to consider preventive medication.^18^ Patients’ knowledge about side effects was mainly based on the experiences of family and friends, or the media.^18,34^

#### Health professional attitudes towards prescribing preventive drugs

##### Addressing patient concerns and information

Some GPs discussed patients’ reluctance to take statins, explaining that patients are usually worried about side effects, or have concerns based on misinformation. This could complicate the discussion with the patient about preventive drugs.^34^ Patients’ former doctors were mentioned by some GPs as another source of information, especially if their current GP’s advice differed.^34^

##### Duty as a health professional to prescribe

###### Addressing CVD risk

In relation to statin prescribing, one GP explained that guidelines are just a guide and there is no definite predicted CVD risk (QRISK score) threshold above which statins must be prescribed.^34^ Other GPs explained that patients with predicted 10-year CVD risk (QRISK) of 15–20% (just below the guideline threshold of 20%) need to consider the option of statins.^34^ GPs further highlighted that they like to start the conversation of heart health early, when patients are borderline. GP views about recommending statins were more consistent in patients with predicted ten-year CVD risk (QRISK) of 20%. They expressed the need to be realistic with higher risk patients.^34^ One GP expressed his certainty that statins were the best approach to address the CVD risk in high-risk patients,^34^ while some GPs discussing cardiovascular preventive drugs expressed their uncertainty about who is truly at risk, related to hard-to-measure factors such as stress.^31^ One GP expressed the view that a polypill should be only for patients with risk factors such as family history of CVD.^32^ Another saw the use of a polypill for primary prevention as allowing better coverage of the population and ultimately reducing cardiovascular risk.^32^

###### Following guideline recommendations

Some GPs expressed their sense of responsibility to follow the guidelines and their trust in the knowledge, expertise, and evidence reviews of guideline authors.^31^ This was also mentioned in relation to the polypill, with one nurse practitioner indicating she would prescribe it if it were endorsed by the Department of Health.^32^

###### Financial conflicts of interest

Some GPs expressed their concern about the ethics of financial incentives in the Quality and Outcomes Framework and the General Medical Services contract creating pressure to prescribe statins and, potentially, to overlook patient preferences.^18^

##### Uncertainty about preventive drug prescribing

###### Health professionals’ personal views on primary prevention

GPs explained they prioritised symptom-relieving drugs over preventive drugs.^31^ One GP indicated he, himself, would only take drugs for symptom relief and not for prevention, but that he considered the decision to prescribe preventive drugs to his patients to be completely different.^18^ Concerns about wastage of taxpayer money were also mentioned by some GPs.^18^

###### Polypill specific issues

Most health professionals discussing the polypill had some understanding of what it would be used for but not of whether it is safe or effective for primary prevention.^32^ Nevertheless, they recognised the practicality of one pill instead of multiple medications, and the potential to improve adherence.^32^ The inability to titrate the dosage made them more reluctant to consider prescribing and questions arose about the effects of combining multiple medications into one pill.^32^

##### Recognising consequences of prescribing

###### Enabling patients to be complacent about leading a healthy lifestyle

GPs shared their concern that prescribing drugs for primary prevention would make patients less likely to change unhealthy habits by providing *‘* *a false sense of security* *’* about lifestyle choices and described drugs as *‘* *an easy way out* *’*.^31,32^ GPs explained that they would discuss all options in relation to cardiovascular prevention with their patients.^18,31,34^ Some expressed preference to start with lifestyle modification but would try to determine the patients’ treatment preference.^18,34^

###### Unnecessary medicalisation of healthy people

Health professionals felt that prescribing preventive drugs labels healthy individuals with a diagnosis, and that patients should be educated about how to maintain their health instead of committing them to taking medication.^31,32^ In relation to the polypill, a nurse practitioner said he doesn’t believe in medicating people, and that patients should learn how to live and deal with the ageing process rather than taking a tablet.^32^

##### Personalised treatment

###### Considering personal factors and risk

GPs expressed the importance of personalising treatment, and that ethnicity and family would affect their decision to accept treatment.^18^ One GP highlighted the importance of personalising treatment by referring to the patient as the ’*patient in front of me’*.^31^ When discussing statin prescribing, GPs explained the need to modify multiple risk factors.^34^ The need for individual dose titration was also mentioned in relation to the polypill.^32^

###### Quality of discussion

Most GPs mentioned the importance of their discussion about preventive treatment and how they attempt to involve the patient in the decision-making process.^31,34^ GPs who discussed attitudes about prescribing statins explained that the quality of the discussion is dependent on the patients’ attitudes about the drug and the level of agreement between the patient and the practitioner.^34^ Some said a comprehensive discussion and explanation of benefits and risks would increase the patient’s trust in their health professional.^34^

## Discussion

### Summary

Patients’ willingness to accept preventive drugs was related to whether they believed the benefits outweigh the risks; having the option to try lifestyle modification before agreeing to take drugs; their family and friends’ experiences, specifically negative experience such as heart attacks that prompt an action to take preventive drugs; and their trust in their health professional advice. Patients had their own individual preferences, including lifestyle modifications or alternative medicine. Health professionals’ willingness to prescribe was related to whether they could address their patients’ concerns and preconceived ideas about preventive drugs. Health professionals also considered it their duty to address risk and follow the guideline recommendations. Some health professionals had views about addressing the calculated CVD risk (QRISK) in patients who are borderline high-risk. They aimed to address the risk as early as possible in high-risk patients and assess any possible patient characteristic that affects CVD risk such as family history and ethnicity. A consistent theme among health professionals was the importance of communicating with patients, discussing their CVD risk, and personalising treatment options.

### Strengths and limitations

This study undertook a wide search for published studies including grey literature, used predefined inclusion criteria, and study selection was conducted by two independent reviewers. NVivo (version 12) was used to facilitate data analysis and theme construction. Two independent reviewers conducted study selection, quality assessment, and data analysis. To ensure minimum subjectivity, each step of the review was discussed by two reviewers, and an advisory team was consulted on the emergent themes. Unlike previous reviews, this study focused on drug initiation for primary prevention, and combined the attitudes of health professionals and patients to give a better understanding of both sides involved in the decision-making process. This review included any potential health professionals who could prescribe cardiovascular drugs and all drugs that could be prescribed for primary prevention were considered, regardless of country. However, there were some limitations. Although the aim was to review studies that assess attitudes for primary prevention, the number of studies eligible to include was low. Despite the authors' efforts, studies that did not explicitly explain that the results were applicable to the context of primary prevention may have been missed. This study's findings may be limited by the low number of nurse practitioners included in the studies, and the lack of details about the type of cardiovascular drugs considered in some studies.

### Comparison with existing literature

Other systematic reviews of patients’ and health professionals’ attitudes towards statins for primary and secondary prevention of CVD reported similar findings to this study's.^22,23^ In a systematic review about patients attitudes towards statins, patients were accepting of statins if they believed that the drug benefited them by potentially preventing CVD and prolonging their lives.^23^ The review discussed barriers to accepting statins such as fear of debilitating side effects or perpetual dependency.^23^ Concerns about over-medicalisation were also reported by GPs.^22^ This review built on the findings of existing research by identifying patients’ and health professionals’ concerns about complacency with trying a healthy lifestyle and over-medicalisation. Patients’ were also concerned about side effects and dependency. Another systematic review addressing initiation of and adherence to cardiovascular preventive drugs highlighted that the patient–health professional relationship affects the decision to start preventive drugs.^17^ The authors found that both patients and health professionals expressed a preference for lifestyle changes and that health professionals try to avoid medicalising healthy patients.^17^ This study's findings suggest that patients preferred to try lifestyle changes before accepting drugs, and that health professionals also prefer lifestyle changes, but they will first consider the patient risk factors and determine patient treatment preferences. This review found that patients trust in health professionals is a key element in accepting treatment. Previous studies refer to trust as an essential factor to an effective patient–health professional relationship.^35–37^ Patients’ trust in their health professional was associated with improvements in lifestyle choices and blood pressure control.^38^

### Implications for research and practice

This review revealed attitudes that could potentially lead patients to accept preventive drugs and health professionals to prescribe them. However, it highlighted research and practice areas where action is needed. Patients’ trust in their health professional advice influenced their willingness to accept preventive drugs. Therefore, future research exploring patients views on what makes a health professional trustworthy can provide health professionals with tools to utilise when establishing a relationship with their patients. In addition, future research exploring cardiovascular drugs should aim to make clear differentiation between evidence applicable for primary or secondary prevention. Compared to the number of GPs included in this review, the number of practice nurses included was low. Thus, researchers should maximise their efforts, when possible, to include relatively equal numbers of GPs and nurse practitioners when investigating issues relevant to clinical practice. This review found that a health professional’s recommendation is an influential factor for drug initiation. This suggests the need for health professionals to ensure they establish a trusting relationship with patients where concerns are addressed, and treatment options are discussed thoroughly. Information provided in the treatment consultation needs to be personalised to equip the patient in making an informed, shared decision. Information regarding drug efficacy for primary prevention, potential side effects, and possible health benefits gained from CVD risk reduction need to be communicated clearly to the patient. Personalising treatment to fit the patients’ own priorities while ensuring that their CVD is addressed could lead to drug initiation for primary prevention. These clinical considerations can be facilitated by research that further investigates the patient–health professional relationship and the communication methods that would apply to addressing patients with no underlying medical conditions.
